# Composition, Influencing Factors, and Effects on Host Nutrient Metabolism of Fungi in Gastrointestinal Tract of Monogastric Animals

**DOI:** 10.3390/ani15050710

**Published:** 2025-03-01

**Authors:** Xiaofeng Deng, Hua Li, Aimin Wu, Jun He, Xiangbing Mao, Zhaolai Dai, Gang Tian, Jingyi Cai, Jiayong Tang, Yuheng Luo

**Affiliations:** Key Laboratory for Animal Disease-Resistance Nutrition of Ministry of Education of China, Key Laboratory for Animal Disease-Resistance Nutrition and Feed of Ministry of Agriculture of China, Key Laboratory of Animal Disease-Resistant Nutrition of Sichuan Province, Engineering Research Center of Animal Disease-Resistance Nutrition Biotechnology of Ministry of Education of China, Animal Nutrition Institute, Sichuan Agricultural University, Chengdu 611130, China; dxf1693465389@gmail.com (X.D.); spbee1757@163.com (H.L.); wuaimin0608@163.com (A.W.); hejun8067@163.com (J.H.); acatmxb2003@163.com (X.M.); daizhaolai@cau.edu.cn (Z.D.); tgang2008@126.com (G.T.); jycai2004@aliyun.com (J.C.); tangjiayong@sicau.edu.cn (J.T.)

**Keywords:** non-ruminant animal, gut, fungal community, contribution

## Abstract

Intestinal fungi, collectively referred to as mycobiota, constitute a small (0.01–2%) but crucial component of the overall intestinal microbiota. While fungi are far less abundant than bacteria in the gut, the volume of an average fungal cell is roughly 100-fold greater than that of an average bacterial cell. They play a vital role in nutrient metabolism and maintaining intestinal health. The composition and spatial organization of mycobiota vary across different animal species and are influenced by a multitude of factors, including age, diet, and the host’s physiological state. At present, quantitative research on the composition of mycobiota in monogastric animals remains scarce, and investigations into the mechanisms underlying their metabolic functions are also relatively restricted. This review delves into the distribution characteristics of mycobiota, including *Candida albicans*, *Saccharomyces cerevisiae*, *Kazachstania slooffiae*, in monogastric animals, the factors influencing their composition, and the consequent impacts on host metabolism and health. The objective is to offer insights for a deeper understanding of the nutritional significance of intestinal fungi in monogastric animals and to explore the mechanisms by which they affect host health in relation to inflammatory bowel disease (IBD), diarrhea, and obesity. Through a systematic evaluation of their functional contributions, this review shifts our perception of intestinal fungi from overlooked commensals to key components in gut ecosystem dynamics, emphasizing their potential in personalized metabolic control regulation and the enhancement of disease prevention and treatment strategies.

## Simple Summary

Although gut fungi account for only 0.01–2% of the gut microbiota, they play a crucial role in nutrition metabolism and gut health. The composition of gut fungi varies significantly across different animal species and gut regions and is influenced by factors such as diet, age, and host physiology. Despite their importance, research on the fungal communities (mycobiota) in monogastric animals remains limited, particularly regarding species-level diversity and functional mechanisms. This review highlights key fungal species, such as *Candida albicans* and *Saccharomyces cerevisiae*, and discusses their distribution across different animal species and gut regions. Additionally, we explore the factors influencing gut fungi and describe how they impact host gut metabolism and health, including their associations with inflammatory bowel disease, obesity, and diarrhea. While advanced technologies like metabolomics have begun to reveal fungal functions, most studies show correlations rather than causal relationships. Future research should focus on elucidating the mechanisms underlying fungal metabolism and immune interactions to translate these findings into targeted therapeutic strategies. By shifting gut fungi from overlooked residents to potential therapeutic targets, this work paves the way for personalized strategies to improve animal gut health and disease management.

## 1. Introduction

In monogastric animals, the intestine functions as the principal site for digestion and nutrient absorption. It also plays a crucial role in the organism’s immune defense via physical and chemical barriers, the innate immune system, the adaptive immune system, and interactions with the gut microbiota [1]. The intestinal epithelial surface is densely populated with five major families of pattern recognition receptors (PRRs): Toll-like receptors (TLRs), NOD-like receptors (NLRs), RIG-I-like receptors (RLRs), C-type lectin receptors (CLRs), and AIM2-like receptors (ALRs). These receptors constitute a critical component of the innate immune system [2]. The microbiome, including fungi, can interact with these innate immune receptors, activating host-protective immune pathways related to the integrity of the epithelial barrier. However, it can also trigger reactions that lead to events associated with IBD [3]. Moreover, a broad spectrum of microbial metabolites, such as short-chain fatty acids (SCFAs) and bile acids, can initiate immune signals to regulate the development, maturation, and maintenance of immune function and homeostasis [4,5]. The intestine harbors a diverse microbial ecosystem, predominantly composed of bacteria and archaea, along with viruses, fungi, and a small proportion of other eukaryotic microorganisms. These symbiotic microorganisms are integral to nutrient metabolism and intestinal homeostasis, leading to microbiota–host interactions commonly referred to as “cross-talk” [6,7]. Research predominantly focuses on the composition and function of bacteria within the animal intestine. However, the mycobiota, or fungal community in the intestine [8], receives comparatively little attention due to its lower abundance relative to bacteria. Accurate measurements of fungal content in the intestines of monogastric animals remain lacking, but current research findings demonstrate that mycobiota account for merely 0.01–2% of the total microbiota within the intestines [3,9,10,11,12,13]. Although fungi are significantly less abundant than bacteria in the gut, the volume of a typical fungal cell is approximately 100 times larger than that of a typical bacterial cell [14] and may still play a vital role in gut health and nutritional function [15]. A plethora of studies have demonstrated that fungi, including *Candida albicans* and *Saccharomyces cerevisiae*, exert a substantial influence on the intestinal health of monogastric animals [16,17,18,19]. Additionally, *Malassezia restricta*, *Meyerozyma guilliermondii*, *Thermomyces*, and *Piromyces* are notably associated with obesity, diarrhea, alcoholic liver disease, and other diseases [20,21,22,23]. Advances in sequencing technology enhance the efficiency and accuracy of mycobiota analysis [24,25,26]. Recent innovations such as environmental metabarcoding (EMT) and spatial metatranscriptomics (SMT) enable rapid documentation of previously unknown fungal diversity and facilitate comprehensive analyses of the functional metagenomics, transcriptome, and microbiome of the host at high resolution [27,28,29]. These developments pave the way for a thorough understanding of the changes in mycobiota composition and distribution in the intestines of animals, their influencing factors, and the mechanisms by which fungi contribute to the host’s physiological activities, including metabolism and immunity. This review endeavors to provide a comprehensive summary of the distribution patterns of fungi among diverse monogastric animal species and within various segments of the animal gut. It also delves into the factors that shape the composition of intestinal fungi in monogastric animals, as well as the pivotal roles played by fungi in nutrient metabolism and the maintenance of gut health, revealing the gaps in mechanistic research.

## 2. Analytical Methodologies for Gut Mycobiota

Historically, research on gut fungi predominantly depended on culture-based methods, which were developed from the late 19th to early 20th century. These methods employ selective media and specific growth conditions to isolate and identify fungal species according to their morphological and biochemical characteristics [30]. Although culture-based approaches allow for the cultivation of viable microorganisms, their application in studying gut microbial diversity is restricted because many gut fungi cannot be cultured under laboratory conditions. Additionally, these methods frequently lead to an underestimation of low-abundance fungi or those that require symbiotic interactions, thus introducing bias into the study outcomes.

To overcome these limitations, with the advent of molecular biology techniques in the early 21st century, ITS (Internal Transcribed Spacer) sequencing became popular in gut microbiota research. The ITS region, a hypervariable segment of ribosomal DNA (rDNA), serves as a universal DNA barcode for fungal taxonomy and phylogenetics due to its high discriminatory power across diverse fungal lineages [24]. Compared with culture-based methods, ITS sequencing offers a more comprehensive picture of fungal community composition. Nevertheless, challenges like PCR amplification bias, incomplete reference databases, and sequencing errors still exist [25,30]. While the ITS regions are effective for most genera belonging to phylum Basidiomycota, their resolution is limited in certain Ascomycota taxa (e.g., *Alternaria*, *Aspergillus*, and *Penicillium*) [31]. Despite these drawbacks, ITS sequencing remains a fundamental technique for analyzing gut fungal diversity.

Recent advancements in high-throughput sequencing and spatial omics have introduced advanced methodologies such as Environmental Metabarcoding (EMT) [32] and Spatial Metatranscriptomics (SMT) [28], which have significantly enhanced our understanding of gut fungal ecology and functionality.

EMT combines high-throughput sequencing with environmental DNA (eDNA) markers such as ITS or fungal 18S rRNA genes to non-invasively evaluate species richness across ecosystems [32]. EMT provides higher resolution and sensitivity than traditional ITS sequencing, enabling the detection of rare or hard-to-detect fungi, minimizing methodological biases, and facilitating large-scale data analysis through bioinformatics [32]. However, factors such as environmental contamination and laboratory artifacts can undermine the accuracy of abundance quantification by eDNA metabarcoding [33]. Robust bioinformatics pipelines and statistical validation are essential to ensure data reliability [34].

SMT is an emerging technology that integrates spatial resolution with transcriptomic profiling. By using multimodal arrays including 16S, 18S, and ITS at 55-μm resolution, SMT can simultaneously map host transcriptomes and microbial gene expression within tissue sections [28]. Different from EMT, SMT provides spatially resolved insights into microbial distribution, host–microbe interactions, and context-dependent gene expression dynamics. It also integrates with multi-omics approaches for a holistic analysis [35]. Nevertheless, SMT confronts technical and economic challenges, including strict sample preparation requirements, risks of RNA degradation, and increased susceptibility to environmental contamination. Mitigating these issues requires spatial filtering of sequencing reads to distinguish biological signals from noise [28].

## 3. Composition and Distribution of Mycobiota in Monogastric Animals

Fungi are ubiquitously present in the intestines of monogastric animals, encompassing mice [36], swine [14], giant pandas [37], dogs [38], cats [38], equines [39], and numerous other herbivorous species [40,41] (Table 1). The composition of the mycobiota exhibits considerable variation among different animal species [42], intestinal segments [43,44], and even between the mucosa and lumen within the same intestinal segment [7].

### 3.1. Composition of Intestinal Fungi in Different Animals

#### 3.1.1. Mouse

Similar to pigs, Ascomycota [45,46,47] and Basidiomycota [45,46] are the most abundant fungal phyla in the mouse intestine, followed by Chytridiomycota and Zygomycota [13]. At the genus level, significant differences arise due to genetics and diet. However, yeasts from the *Candida* and *Saccharomyces* genera predominantly occupy the mouse intestine [48]. It is reported that the intestine of Advanced Intercross Lines (AILs) mice harbors the highest abundance of *Penicillium* spp. [46], followed by *Aspergillus* spp. [46] and *Candida* spp. [46]. At the species level, *Candida albicans* [13,48] and *Saccharomyces cerevisiae* [48] typically demonstrate higher abundance in the mouse intestine [59,60].

#### 3.1.2. Pig

The composition of mycobiota displays significant variability among various pig breeds [49,51]. Numerous studies pinpoint that Ascomycota [49,50,51], Basidiomycota [50,51], Mucoromycota [49,52], Microsporidia [49], Mortierellomycota [49,52], and Chytridiomycota [49,52] as the dominant fungal phyla within the intestinal tracts of pigs, including DLY (Duroc × Landrace × Yorkshire), Tibetan, and Taoyuan pigs [47,49,51,52,61]. Among these, Ascomycota stands out as the most abundant [11,45,51,52,53], while Rozellomycota is notably more prevalent in the intestinal tract of Tibetan pigs [50]. At the genus level, fungal species exhibit considerable diversity. *Kazachstania*, *Mucor*, *Anaeromyces*, *Piromyces*, and *Neocallimastix* are the primary genus-level fungi in the intestines of three pig breeds, including Taoyuan, Duroc, and crossbred Xiangcun. Furthermore, *Nakaseomyces*, *Zygosaccharomyces*, *Aspergillus*, and *Ganoderma* display notable variation among these three breeds and exhibit vertical transmission [49]. In contrast, the dominant genera in the intestinal tract of DLY pigs encompass *Naganishia*, *Rhodotorula*, *Fusarium*, *Mortierella*, and *Candida* [52]. The fungal flora of wild boars is more intricate compared to domestic pigs, with *Kazachstania*, *Saccharomyces*, and *Aspergillus* being more abundant [62]. At the species level, *Kazachstania slooffiae* [11], *Kazachstania telluris* [47], and *Kazachstania bovina* [11] are more prevalent in the intestines of pigs, including wild boars. Notably, *K. slooffiae* is found to be the most abundant fungal species in weaned piglets and fattening pigs of DLY, Shaziling, and Ningxiang breeds [53].

#### 3.1.3. Giant Panda

Despite the scarcity of studies on the mycobiota of giant pandas, it is reported that the phyla Basidiomycota [37,54], Ascomycota [37], and Glomeromycota [37] dominate the fungal community of these animals, maintaining a stable abundance throughout their growth [37,54,63,64]. *Sordariomycetes* (~40.0% [37]), *Saccharomycetes* (~16.7% [37]), and *Leotiomycetes* (~10.7% [37]) are the most prevalent classes in the intestines of giant pandas. Although certain fungal genera in the intestines may exhibit increased abundance due to various factors, the proportion of dominant genera does not exceed 25%, suggesting remarkable stability that may be attributed to the panda’s self-protection mechanisms [55]. Consequently, the dominant genera identified in the intestines of different individual giant pandas can vary significantly. Specifically, the identified dominant genera include *Fusarium* [37], *Brettanomyces* [37], and *Oidiodendron* [37], which differ considerably from the findings of another research, in which *Montagnulaceae* [55] and *Trimmatostroma* [55] are reported as the dominant genera. Furthermore, *Fusarium oxysporum*, *Fusarium proliferatum*, and *Brettanomyces custersianus* are reported as the most abundant species [37].

#### 3.1.4. Dog and Cat

In cats, the predominant phyla detected are Ascomycota [38] and Basidiomycota [58]. In dogs, Ascomycota [57], Basidiomycota [57], Glomeromycota, and Zygomycota are identified. *Nakaseomyces* [38] emerges as the most abundant fungal genus in dogs. In cats, *Saccharomyces* [38], *Aspergillus* [38], *Peniophorella* [58], and *picha* [58] are the dominant genera. Moreover, *Candida castellii* [38], *Candida natalensis* [57], and *Catenulostroma abietis* [57] exhibit the highest abundance in dogs.

#### 3.1.5. Herbivorous Monogastric Animals

Within the gastrointestinal mycobiomes of equids (*Equus* spp.), anaerobic fungi of the phylum Neocallimastigomycota, especially representatives from the genus *Caecomyces* [39], along with an uncultivated genus named *AL1* [39], are the dominant taxa across multiple equid lineages. These lineages include donkeys (*Equus asinus*), ponies (*Equus ferus caballus*), horses (*Equus ferus caballus*), their hybrids (horse × donkey, pony × donkey), and zebras (*Equus quagga*) [65,66,67]. Notably, Neocallimastigomycota shows extraordinary dominance (>99.9% relative abundance) in the donkey intestinal ecosystem [67], while its prevalence in horses varies significantly across different studies [20,41]. Furthermore, Ascomycota [20,41], Mucoromycota [41], and Basidiomycota [20,41] are also quite prevalent in horses [20,41]. At the class level, *Leotiomycetes* (~24.44% [41]) and *Pezizomycetes* (~44.74% [41]), which belong to the Ascomycota phylum, dominate the intestinal fungal community of horses. At the family level, *Pilobolaceae*, *Ascobolaceae*, *Davidiellaceae*, and *Neocallimastigaceae* are the predominant families in horses [41]. At the genus level, *Cladosporium* and *Cryptococcus* have the highest abundance in the intestines of Mongolian horses [15], and a novel uncultivated taxon (*SK3*) seems to be unique to the donkey microbiota [65].

In rabbits, Ascomycota [41] and Basidiomycota [41] are the predominant fungal phyla in the intestines. At the class level, *Leotiomycetes* (~47.06%) [41] and *Pezizomycetes* (~24.66%) [41], which belong to the Ascomycota phylum, dominate the intestinal fungal community. At the family level, *Thelebolaceae* is dominant [41]. At the species level, *Cyniclomyces guttulatus* [21] is the most abundant species [21].

The mycobiomes of proboscideans show phylum-level convergence with those of other herbivores. In African bush elephants (*Loxodonta africana*), Ascomycota and Basidiomycota are the principal components. At the genus level, *Alternaria*, *Aureobasidium*, *Didymella*, and *Preussia* are the most prevalent, and there are some individual-specific taxa, such as *Aureobasidium*, *Botryotrichum*, *Camarosporium*, *Lasiobolus*, and *Zygosaccharomyces* [40]. The representative species are *Trichoderma aureoviride* and *Fusarium equiseti*, two fungi with significant cellulose-decomposition ability, which are isolated from Asian elephants (*Elephas maximus*) [56].

### 3.2. Distribution of the Mycobiota Across Various Segments of the Gastrointestinal Tract (GIT)

**Figure 1:**
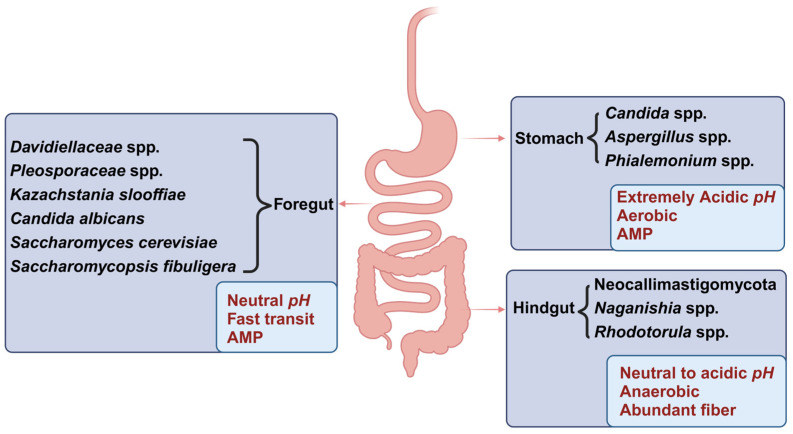
Predominant fungal taxa at different levels in animal intestinal segments.

Living systems across diverse dimensions are deeply influenced by their surrounding environments. Similar to how macroscopic organisms carefully select their habitats, microbial colonization within the GIT also exhibits distinct selectivity [68]. The environments of different segments within the GIT vary significantly in terms of pH [69,70], oxygen concentration [3], coeliac flow rate [71,72], bile acid concentration [73], and gastrointestinal secretions, such as antimicrobial peptides (AMPs) [74]. These differences contribute to the differential colonization patterns of fungi in various gastrointestinal segments [64,75], highlighting their respective suitability for fungal growth (Figure 1). This spatial variability exerts a considerable impact on the host’s metabolism and gastrointestinal health [76].

*pH* level is a pivotal factor in determining the distribution of fungi within the intestine. The regulation of intestinal *pH* is mainly governed by the exchange of Cl^−^/HCO3^−^ and Na^+^/H^+^ in the epithelial cells of the GIT, as well as by the concentrations of SCFAs and bile acids [77]. Bile acids also play a crucial role in modulating fungal distribution by altering intestinal *pH* [73]. The extremely low *pH* of the stomach, ranging from approximately 3.74 to 4.24 in mice [70], is generally considered unsuitable for the survival of most fungi. However, certain fungi, like *Candida*, demonstrate remarkable adaptability through the up-regulating or down-regulation of specific genes. For example, the expression of the *Rfg1* gene regulates the acidic *pH* response via the Rim101-Phr1 pathway [78]. Additionally, fungal genera such as *Aspergillus* and *Phialemonium* can colonize the stomach despite its harsh low-*pH* conditions, as they possess the ability to secrete AMPs [79,80]. *Candida* spp. can promote the digestion of dietary starch [46] and play an important role in initiating glycolysis [81]. *Aspergillus* spp. [82] and *Phialemonium* spp. [83] are closely associated with lipid metabolism. These fungi may potentially facilitate the digestion and metabolism of carbohydrates and fats in the stomach.

The oxygen concentration in various intestinal segments can be influenced by a range of metabolites. For instance, elevated levels of butyrate in the hindgut restrict the diffusion of oxygen from the intestinal epithelium into the lumen, thus maintaining anaerobic conditions in that specific region [44]. Consequently, variations in oxygen content among different intestinal segments have a significant impact on the distribution of aerobic or facultative anaerobic fungi [84]. Since the stomach lumen is considerably aerobic [60], the primary colonizers in the jejunum of pigs are *Kazachstania slooffiae* and other predominantly aerobic or facultative anaerobic fungi [53,84]. *Aspergillus* and *Phialemonium* are also capable of adapting to the high oxygen concentration in the stomach [79,80]. *K. slooffiae* can promote glycolysis in intestinal epithelial cells (IECs) [83] and degrade a high proportion of peptides [85], suggesting its potential role in enhancing carbohydrate and protein metabolism in the jejunum. The foregut of Mongolian horses is dominated by phyla Basidiomycota (~60.02%) [15] and Ascomycota (~36.19%) [15], especially aerobic families *Davidiellaceae* and *Pleosporaceae*, with an enrichment of various pathogenic fungi. For instance, *Cladosporium*, belonging to *Davidiellaceae*, is related to nonalcoholic fatty liver disease (NFLD) [15], IBD [86], and even HIV [87], and *Alternaria alternata*, belonging to *Pleosporaceae*, can alter the structure and function of colonic epithelial cells through toxins AOH and ATX-II [88]. In contrast, the hindgut is enriched with the Basidiomycota (~22.91%) and Neocallimastigomycota phyla (~53.84%) [15], including numerous anaerobic fungi of the *Neocallimastigaceae* family and other fungi associated with cellulose digestion [15].

The availability of nutrients varies across different gastrointestinal segments. The stomach provides limited nutritional sources for fungi, while the small and large intestines contain a diverse range of food compounds and plant fibers, facilitating fungal involvement in nutrient digestion. Neocallimastigomycota is enriched with hemicellulases (particularly GH10) and polysaccharide deacetylases [89], and Basidiomycota produces a variety of lignocellulolytic enzymes [90], enabling the efficient utilization of recalcitrant substrates such as lignocellulose and hemicellulose in the hindgut, thereby providing nutrients and energy to the host. The hindgut is rich in substrates required for Neocallimastigomycota fermentation, which explains the high abundance of Neocallimastigomycota phyla, including the *Neocallimastigaceae* family and other fungi associated with cellulose digestion in the hindgut of equids [15]. The abundance of the Basidiomycota (2.93 → 91.25%) and Mucoromycota (0.004 → 0.43%) phyla, as well as the *Naganishia* (2.10 → 80.20%) and *Rhodotorula* (0.32 → 9.71%) genera increase progressively from the stomach to the hindgut of pigs [52]. Since *Naganishia albida* [91] and *Rhodotorula* spp. [92,93,94] have an excellent ability to degrade various nutrition resources, they may play a nutritional role similar to that of Neocallimastigomycota, which is abundant in the equine hindgut.

In addition to *pH*, oxygen, and substrate availability, which are the three primary influencing factors, intestinal motility, chyme flow rate, local immune responses, and intestinal inflammation also jointly affect the distribution of gut fungi. Due to stronger peristalsis and faster chyme flow in the small intestine, only rapidly growing fungi or those capable of colonizing the mucosa, such as *Candida albicans*, *Saccharomyces cerevisiae*, and *Saccharomycopsis fibuligera*, can thrive in this environment [7]. The gastric lumen produces peptides with strong antimicrobial properties [95], while Paneth cells in the small intestine secrete PC-PYY, an AMP, which reduces the abundance of opportunistic pathogens like *C. albicans* in the small intestine [74]. SCFAs, the main metabolites of microbial fermentation, serve as a primary energy source for intestinal cells, helping maintain normal intestinal function, inducing immunomodulatory effects to prevent inflammation and microbial infections, and are crucial for maintaining homeostasis [44]. Bile acids can also modulate fungal distribution by influencing host intestinal immunity [73]. Intestinal inflammation, such as in IBD, can alter fungal composition (decreasing the ratio of Basidiomycota to Ascomycota [86]), promote the proliferation of opportunistic pathogenic fungi like *C. albicans*, and reduce the abundance of certain fungi, such as *S. cerevisiae* [7].

Consequently, fungal abundance in the small intestine is generally lower than in the large intestine [76] due to several factors that are not conducive to microbial colonization. These include a higher *pH*, elevated oxygen content, high concentrations of bile acids, the presence of AMPs, the inhibitory effects of immune proteins [74,96,97], and a faster flow rate of digesta. In the large intestine, the environment is significantly more conducive to fungal survival. The neutral-to-acidic *pH*, very low salt content, a slow flow rate of digesta, abundant supply of fiber, and the protective mucus layer of the epithelial cells synergistically create an excellent habitat for fungi, particularly anaerobic fungi [8,44,77,80,84]. However, upon summarizing previous research, it becomes clear that we cannot conclusively determine the enrichment of most specific fungi in different intestinal segments of animals, as this is significantly influenced by various factors such as species, age, diet, and environment. It has been found that the fungal abundance and diversity in the hindgut of Mongolian horses are notably higher compared to the foregut [15]. In contrast, it has been reported that the abundance of fungi in the duodenum (1.5 ± 1.00 log CFU/g) and small intestine (1.5 ± 1.00 log CFU/g) is higher than that in the caecum (0.8 ± 1.50 log CFU/g) and colon (0.5 ± 1.00 log CFU/g) of a rabbit [98]. Another study in mice reports that the highest fungal diversity is observed in the jejunum [99].

### 3.3. Distribution of the Mycobiota in Intestinal Lumen and Mucosa

Significant variations in the intestinal environment exist not only between different intestinal segments but also locally between the lumen and the mucosa [7,100].

Fungi are abundant within the lumen of all intestinal segments, and some of them possess the capability to stably adhere to epithelial cells, thereby colonizing the unique environment of the intestinal mucosa [7]. Some examples of mucosal-colonizing fungi include *Candida albicans*, *Saccharomyces cerevisiae*, *Saccharomycopsis fibuligera*, *Aspergillus amstellodamii*, *Cladosporium cladosporioides*, and *Wallemia sebi*, which primarily colonize the lumen [7,101,102]. Additionally, SCFAs impact the growth and adhesion of fungi, such as *Pichia kudriavzevii*, that colonize the mucosa and attach to IECs [103].

## 4. Factors Influencing the Composition of the Intestinal Mycobiota

Although the mycobiota generally exhibit greater stability compared to bacterial communities [104], not all fungi are capable of stably colonizing the host intestine, and the abundance of most fungal species is influenced by a multitude of factors [54]. The composition of the mycobiota is affected by host age [6,52], diet [23,48], host physiological status [17,104], environmental conditions [105], and antibiotic use [106]. Among these factors, age and dietary composition have the most profound impact on the mycobiota [76,104].

### 4.1. Age

In addition to individual differences, considerable variations in the mycobiota are observed among animals of different ages [104,107,108], especially during the early stages of growth [53,59]. Especially during the early stages of growth, a developing immune system and immature bacterial community could allow fungi to have a greater variation [53,59].

The extent to which age-induced changes in fungal composition outweigh individual differences remains uncertain. The diversity of porcine intestinal fungi increases with age [53]. A taxonomic study of intestinal fungi in Congjiang piglets further indicates that fungal abundance also rises with age, showing a significant increase in *Candida*, *Aspergillus*, *Cladosporium*, and *Simplicillium*, while *Kazachstania* and *Aureobasidium* decrease [47].

Numerous studies suggest that the observed effect of age on the mycobiota is largely superficial, with the underlying cause being substantial alterations in diet, living environment, intestinal development, and other physiological factors that occur with aging. These changes lead to greater instability in the mycobiota compared to the bacterial community [75,80,105]. Notably, during the weaning period, significant dietary shifts result in pronounced changes. It is reported that the abundance of fungi in piglet feces varies significantly at both the phylum and family levels, with *Kazachstania slooffae* being particularly notable [105,109]. In contrast to pigs, the transition from breast milk to bamboo in giant pandas is markedly more drastic, resulting in more pronounced, complex, and enduring changes in intestinal fungal composition that can last approximately 1.5 to 2 years [110]. From the pre-weaning to the post-weaning stage, the composition of anaerobic fungi in the feces of donkey foals experiences substantial alterations. Prior to weaning, the abundance of unclassified order and genus of *Neocallimastigales* was higher than those in other periods. During the weaning period, the abundance of *Orpinomyces* increases markedly [67]. Therefore, it is evident that the weaning period represents a unique “window” during which the mycobiota undergoes significant transformations [6].

### 4.2. Diet

Diet has a profound impact not only on the metabolic functions of the host’s digestive system directly but also on the composition and metabolic activities of the mycobiota in both the short and long term. This influence facilitates the host’s adaptation to environmental changes, as the mycobiota exhibits greater sensitivity to alterations in dietary composition compared to bacteria [23]. Moreover, fungal species belonging to the same genus are frequently co-regulated by dietary factors [8,46,76,111].

Fungi such as *Aspergillus*, *Saccharomyces*, *Penicillium*, *Candida*, *Cladosporium*, *Picha*, and others from phylum Ascomycota, as well as *Malassezia* from phylum Basidiomycota, are prevalent in the animal gut [8,61]. However, many of these fungi are also ubiquitous in food [104], entering the digestive system through the diet and influencing the composition of the mycobiota. Only a subset of these fungi, including *Aspergillus*, *Penicillium*, *Saccharomyces*, and *Candida*, are shown to transiently colonize the intestine [7,112], with *Debaryomyces hansenii* and *Saccharomyces cerevisiae* being particularly notable [113]. Prolonged consumption of bamboo leads to the gradual colonization of the giant panda’s intestinal tract by cellulolytic-related fungi such as *Shiraia*, *Aspergillus*, *Penicillium*, and *Trichoderma*, which are predominantly found in bamboo [55].

The composition of dietary fiber has a significant impact on shaping the gut mycobiota, especially those fungi capable of cellulose degradation [15], such as specific species belonging to the Ascomycota phylum [114]. It is demonstrated that diets rich or poor in fiber alter the abundance of fungi at various intestinal levels in mice and giant pandas [49,99]. The presence of cellulose-degrading fungi, including *Aspergillus*, *Penicillium*, and *Trichoderma*, decreased in the intestines of giant pandas when cellulose intake was reduced [55]. The relative abundance of Ascomycota in the intestines of giant pandas fed exclusively on the bamboo-only diet is notably higher compared to those fed solely on formula milk [54]. Conversely, a high-fiber diet can potentially enhance the abundance of intestinal fiber-degrading bacteria, which produce organic acids that inhibit the growth of certain fungi [53]. Thus, the influence of fiber content on fungal composition is multifaceted. Additionally, other dietary factors, such as the percentage of mannan-oligosaccharides, the ratio of amylose to amylopectin, and the levels of non-starch polysaccharides, also substantially influence mycobiota [61]. Furthermore, the solubility of dietary fiber affects mycobiota composition through the production of SCFAs during fermentation [44]. Soluble fiber increases SCFA concentration in the small intestine, while insoluble fiber is primarily fermented in the hindgut, leading to distinct effects [115]. Research shows that carbohydrates, sugar alcohols, and primary bile acids promote the growth, morphogenesis, and metabolic activity of *C*. *albicans*, whereas carboxylic acids, SCFAs, and secondary bile acids exert an inhibitory effect [116,117]. Furthermore, the abundance of fungi is also affected by the protein and fat content in the diet. *Candida* spp. exhibit a negative correlation with diets rich in amino acids, protein, and fatty acids [118]. However, an in vitro study has shown that *C*. *albicans* has a positive association with amino acid uptake [119]. In the murine intestine, the abundances of *S. cerevisiae* (decreased by ~40%), *Fusarium* (~44%), and *Alternaria* (~34%) are significantly negatively correlated with a high-fat diet. Conversely, the abundance of *Aspergillus terreus* and *Candida parapsilosis* (increased by ~75%) display a significant positive correlation [48].

Finally, the proportions of carbon (C), nitrogen (N), and phosphorus (P) in herbivore feces influence the fungal composition. Specifically, fungal diversity diminishes with elevated levels of fecal N and an increased N:P ratio, whereas it augments with higher levels of C and an elevated C:N ratio. Notably, the relative abundance of Ascomycota positively correlates with fecal N, C, and the N:P ratio. Conversely, the abundance of Mucoromycota and Neocallimastigomycota positively correlates with fecal C and C:N ratio [41]. These observations may stem from variations in the dietary proportions of C, N, and P among different animals, ultimately impacting the fungal composition in their feces.

### 4.3. Health Status

The health of the host, especially intestinal health, plays a pivotal role in the modifications of mycobiota that arise from alterations in environmental conditions impacting fungal populations. Especially for livestock at the early growth stage, heat stress can readily trigger a variety of intestinal alterations. These include damage to mucosal epithelial cells, impairment of the intestinal barrier function, elevation of oxidative stress, reduction in immune capacity, and an increase in intestinal permeability to toxins and pathogens. Such changes give rise to diverse intestinal disorders and, in turn, indirectly result in the dysbiosis of the mycobiota [120,121].

When compared to healthy animals, the mycobiota composition in animals suffering from specific diseases exhibits intricate variations. These diseases include IBD [17], diarrhea [122], and obesity [99], each of which has a unique impact on the mycobiota.

IECs possess PRRs, crucial components of the innate immune system that detect pathogen-associated molecular patterns (PAMPs) and damage-associated molecular patterns (DAMPs). These receptors initiate signaling cascades to activate innate immune responses against pathogenic fungi [123]. Among PRRs, CLRs are essential in antifungal immunity [8]. For instance, Dectin-1, a CLR, specifically recognizes β-1,3-glucans, a conserved structural component of fungal cell walls. Upon ligand binding, Dectin-1 recruits the adaptor protein caspase recruitment domain-containing protein 9 (CARD9) to its cytoplasmic domain, triggering the Syk-CARD9 signaling axis. This pathway culminates in the activation of NF-κB, driving the differentiation of Th17 and Th1 cells, which coordinate antifungal immune responses [46,122,124]. Th17 cells exhibit dual roles in intestinal homeostasis. They can enhance mucosal barrier integrity by promoting epithelial repair and antimicrobial peptide secretion [125]. On the other hand, hyperactivation of Th17 cells induces excessive production of pro-inflammatory cytokines, including IL-6, TNF-α, IL-1β, and chemokines such as CXCL1 and CXCL8 by epithelial cells, fibroblasts, and endothelial cells, exacerbating tissue damage and chronic inflammation [18,126,127,128]. Concurrently, Th1 cells contribute to intestinal pathology through the secretion of cytokines such as IL-2, IFN-γ, and TNF-α, which are implicated in the pathogenesis of IBD [123,129]. Thus, the interplay between antifungal immunity and intestinal health is mechanistically intertwined. *C. albicans*, the most abundant commensal fungus in the gut microbiota [17,18,122], dynamically shifts between commensalism and pathogenicity depending on its phenotypic state [130]. Notably, *C. albicans* serves as a central modulator of Th17 responses. It specifically induces epithelial damage and Th17 cytokine release, eliciting robust Th17-driven immunity [96]. Conversely, blockade of IL-17A exacerbates Crohn’s disease (CD), highlighting the critical role of Th17 responses in mitigating *C. albicans*-associated CD pathology [125]. Furthermore, *C. albicans* is a potent inducer of antifungal antibodies, including IgA (mucosal immunity) and IgG (systemic immunity) [10,97]. The production of antifungal IgG is dependent on CARD9, a key innate immune regulator, and CARD9^+^CX3CR1^+^ macrophages [10]. Genetic deficiencies in Dectin-1 or CARD9 impair antifungal IgG responses, heightening susceptibility to invasive fungal infections in animals. These defects are also correlated with dysbiosis of fungal communities and the progression of IBD [8,10,131]. Gut IgA exhibits species-specific reactivity toward fungal communities, with divergent responses observed between systemic and mucosal compartments. Notably, IgA reactivity is markedly reduced against *S. cerevisiae* compared to the robust response elicited by *C. albicans* [132]. Overall, the crosstalk between fungal recognition (via CLRs), adaptive immune polarization (Th17/Th1), and antibody-mediated immunity highlight the intricate balance required for intestinal homeostasis. Dysregulation of these pathways, particularly in the context of *C. albicans* colonization, underscores their relevance to IBD pathogenesis and composition of the mycobiota.

The ratio of Basidiomycota to Ascomycota in the gut is observed to decrease in mice with colitis [86]. This shift is characterized by an elevated abundance of *C. albicans* and a decreased abundance of *S. cerevisiae* [17,18,19]. Research demonstrates that these two fungal species have opposing effects on intestinal inflammation [16]. These findings suggest that changes in mycobiota vary among animals with IBD, with certain fungal strains potentially promoting inflammation. For example, *C. albicans* can damage intestinal macrophages [133,134], which play an important role in the activation and induction of antifungal immunity in the colonic mucosa [127]. Further research into the underlying mechanisms is crucial to identify key fungal strains that could contribute to the mitigation or treatment of IBD. Furthermore, *Malassezia restricta* is identified in the intestines of rabbits suffering from gastrointestinal diseases, a discovery that contrasts with the absence of this fungus in the intestines of healthy rabbits [21].

Certain fungi exhibit close ties to host metabolism, undergoing significant alterations when the host experiences metabolic disorders [135]. *Thermomyces*, *Saccharomyces*, *Rhodotorula*, and *Paecilomyces* are notably associated with obesity [23,111]. Specifically, the abundance of *S. cerevisiae* is lower in obese mice compared to healthy control, whereas *Aspergillus terreus*, a cholesterol-utilizing fungus, shows a higher abundance [48,136]. In mice fed a long-term 4% (*v*/*v*) ethanol-containing diet, the abundance of the symbiotic fungus *Meyerozyma guilliermondii* increases compared to those fed a control diet [22]. These findings suggest that the genus *Candida* may play a key role in metabolic diseases. Thus, further research on the impact of strains like *C. albicans* on host metabolism is imperative to tackle the challenges posed by metabolic disorders.

Diarrhea can markedly decrease the diversity and abundance of fungi in the horse gut, leading to a disruption in the composition of the fungal community. Specifically, Neocallimastigomycota and *Piromyces* are notably more abundant in horses with diarrhea compared to healthy horses [20]. Similarly, in Tibetan piglets suffering from diarrhea, the abundance of Rozellomycota is elevated compared to healthy piglets, along with significant differences in the abundance of *Derxomyces*, *Lecanicillium*, and *Naganishia* [50]. However, in contrast, no differences in fungal composition are observed between healthy piglets and those with diarrhea from a cross between Landrace and Large White pigs [137].

### 4.4. Utilization of Antibiotics

In recent years, the improper application of antibiotics in livestock has elicited significant concern, as it not only contributes to the escalation of bacterial resistance but also potentially adversely affects the diversity of fungi within the gut microbiota, thereby compromising animal health [106,138,139]. Given that many animals are integral components of the human food chain, antibiotic residues may be transmitted to humans through consumption, further exacerbating the proliferation of resistance. Moreover, the inappropriate use of antibiotics can disrupt the equilibrium of the gut microbiome, impairing the host’s immune function and overall health [140]. In response to these potential risks, numerous countries have prohibited the use of antibiotics as growth promoters in animal feed, highlighting the necessity for more responsible and sustainable antibiotic practices [141,142,143].

It is well-established that antibiotics reduce the abundance of bacteria while concurrently increasing the abundance of fungi in the gut [106,144]. Typically, the microbiota returns to its previous state once antibiotic treatment ceases [145]. However, the mycobiota’s response to antibiotics is delayed and prolonged compared to that of bacteria [106]. Despite extensive research on the immediate effects of various antibiotics in the intestinal tracts of different animals, the underlying mechanisms driving these effects remain largely unknown.

The effects of various antibiotics on the mycobiota demonstrate considerable variability, and the mechanisms responsible for these effects are not consistent. A substantial increase in fungal abundance in the intestines of mice treated with cefoperazone is observed [116]. In contrast, a more than 1000-fold increase in the abundance of *Saccharomyces boulardii* in the feces of mice receiving a combination of antibiotics [146]. Another study reports a notable decrease in the abundance of Basidiomycota and Ascomycota in the intestines of mice treated with vancomycin and colistin, respectively [16]. Amoxicillin-clavulanic treatments lead to an increase in the abundance of *Aspergillus*, *Dendrosporium*, *Cladosporium*, and *Valsa*, while potentially inhibiting the overall growth of the mycobiota due to the proliferation of specific bacteria, such as those belonging to family *Enterobacteriaceae* [106]. Furthermore, amoxicillin and macrolides individually increase the abundance of *Candida* and Basidiomycota, respectively, and their combined use results in an increase in *Candida* abundance while decreasing the abundance of *Saccharomyces* [144].

Studies show that the impact of antibiotics on intestinal enteric fungi in subjects is associated with the duration since ingestion, leading to fungal overgrowth and intestinal infections during the early stages of treatment [145]. It is proposed that macrolides may have more prolonged effects on the gut microbiota compared to amoxicillin [144].

Antibiotics exert their influence on mycobiota through a multitude of mechanisms. β-lactam antibiotics, for instance, trigger the release of substantial quantities of peptidoglycan fragments by bacteria, which, in turn, induces the transformation of *C. albicans* from a benign yeast phenotype to a pathogenic mycelial phenotype [147]. Antibiotics can indirectly modify the composition of mycobiota by altering the levels of taurocholic acid (TCA) [73]. Notably, cefoperazone fosters the growth, mycelial development, and gastrointestinal colonization of *C. albicans* by augmenting the levels of growth-promoting metabolites such as carbohydrates, sugar alcohols, and primary bile acids while concurrently decreasing the levels of growth-inhibiting metabolites, including carboxylic acids and secondary bile acids [116]. Beyond their effects on mycobiota composition, antibiotics like vancomycin can impair lymphocyte-dependent IL-17A- and GM-CSF-mediated antifungal immunity in the intestine, thereby increasing the susceptibility of animals to invasive candidiasis, primarily caused by *C. albicans* [73,140].

The widespread use of antibiotics facilitates the evolution of resistance in certain fungi, giving rise to invasive species, particularly *Candida*, which pose considerable threats to animals. As an example, *Candida parapsilosis* displays resistance to 5-fluorocytosine [136]. Recently, studies on fungal resistance have emerged, confirming that the B2 component of a novel BRD4-histone deacetylase (HDAC) inhibitor synergistically interacts with fluconazole. This combination inhibits the growth and morphological transformation of *C. albicans* biofilm-forming mycelium while enhancing fluconazole’s antifungal efficacy in vivo [148].

### 4.5. Interactions Between Microorganisms

#### 4.5.1. Interactions Between Bacteria and Fungi

Fungi and bacteria engage in a diverse array of interactions that can be broadly classified into three primary types: mutualism, commensalism, and competition [149]. For instance, certain bacteria hinder fungal growth by promoting the production of organic acids, while others can stimulate the secretion of antimicrobial substances by specific fungi [53,150]. However, several studies reveal that an impressive 90% of the interactions between fungi and bacteria in the gut of herbivores exhibit a positive correlation, synergistically bolstering the host’s metabolic functions [41]. Specifically, in the duodenum of rabbits, a strong positive correlation is observed between lactic acid bacteria and yeast [98]. The interactions between bacterial and fungal communities in the gut of Mongolian horses may contribute to enhancing their immune system [15]. The influence of bacteria on fungal communities tends to be more potent and enduring than the reciprocal influence of fungi on bacteria. Furthermore, the initial composition of mycobiota benefits positively from the presence of bacteria [59]. Notably, the spatial distribution of *C. albicans* is also influenced by the presence of bacteria [151]. However, it is crucial that bacteria are present in sufficient concentrations to exert a noticeable effect on fungi [106].

The relationship between fungi and bacteria is intricate and multifaceted. While numerous studies explore the correlation between fungal and bacterial abundance in the animal intestinal tract, there remains a significant gap in research regarding the mechanisms underlying mutualistic interactions. At the genus level, *Claviceps*, *Alternaria*, *Davidella*, and *Wallemia* exhibit the highest frequency of interactions with bacteria [46]. *Lactobacillus* spp. and *Bifidobacterium* spp. are negatively correlated with most fungi, including yeasts such as *C. albicans and Rhodotorula mucilaginosa*. However, several other bacterial species exhibit contrasting effects on different fungal species [59]. Additionally, most *Enterobacteriaceae* inhibit the growth of *S. cerevisiae* [106]. The simulation prediction model indicates that certain species of Actinomycetota and Bacteroidota may inhibit *C. albicans*, while members of Firmicutes and Proteobacteria may promote its growth [151]. Conversely, *C. albicans* is shown to increase the relative abundance of Proteobacteria and Cyanobacteria [116]. *Aspergillus* exhibits a negative correlation with SCFA-producing bacteria such as *Butyricoccus*, *Subdolicapsulum*, and *Fusicatenibacter*, whereas *Kazachstania* displays a positive correlation with several bacterial species like *Lactobacillus* [75]. *Hyphopichia* and *Aspergillus* show the highest number of interactions with bacteria in the intestines of weaned piglets, displaying both positive and negative correlations with various bacterial species. Furthermore, competition between bacteria and fungi is observed in the gut of the giant panda [55].

Bacterial–fungal interactions play a crucial role in regulating host metabolism and the development of diseases, which underscores the importance of studying the underlying mechanisms of these interactions. Recent research reveals that *Bacteroides thetaiotaomicron* in mice can foster the growth of *C. albicans* by stimulating the yeast’s colonization of the outer mucus layer [102]. *Enterobacteriaceae*, such as *Escherichia coli*, can facilitate the colonization of yeast-like *C. albicans* and actively contribute to intestinal inflammation [16]. In an in vitro co-culture assay, *Bacillus* species are found to inhibit the production of aspergillic acid and the dipeptide asperopiperazine B by *Aspergillus* while promoting the secretion of Trichodermarin N and Trichodermatide D by *Trichoderma* [150]. Gram-positive bacteria, including *Staphylococcus aureus*, *Staphylococcus epidermidis*, and *Streptococcus pyogenes*, as well as Gram-negative *E. coli*, can bind directly to the leucine-rich repeat (LRR) domain of the adenyl cyclase in *C. albicans* using specific bacterial peptidoglycan (PGN) subunits, thereby promoting its mycelial growth [147]. In contrast, chitin from *Candida glabrata* inhibits the growth of anaerobic bacteria such as *Lactobacillus johnsonii* [152]. A significant positive correlation between *Aspergillus terreus* and various bacterial processes is also identified, including the biosynthesis of bacterial gamma-aminobutyric acid (GABA), methanogenesis, the reductive acetyl coenzyme A (acetyl-CoA) pathway, and beta-oxidation. Furthermore, *Aspergillus* spp. can produce lovastatin, which not only lowers cholesterol but also increases the abundance of bacteria involved in carbon metabolism [48].

In the early stages of a host’s life, fungal and bacterial communities do not exhibit notable mutualistic effects. However, they exert a profound influence on the composition of these communities as the host matures [59]. A study conducted on newborn piglets reveals the absence of bacterial–fungal interactions in their intestinal tract initially, but by day 21, a total of 93 distinct interactions are identified [105].

The majority of systemic immune alterations, encompassing shifts in key immune cell subsets and cytokines, stem from the synergistic impact of bacterial and fungal colonization, suggesting that fungal–bacterial interactions might contribute to the progression of diseases [153]. While fungal colonization alone is inadequate to elicit substantial dextran sulfate sodium (DSS)-induced colitis, the condition necessitates bacterial signaling, and the co-colonization of fungi and bacteria can regulate the intensity of the immune response [59].

Bacteriophages, or phages, which are viruses that exclusively infect bacteria, significantly influence bacterial community dynamics and phenotypic characteristics through either lytic cycles or lysogenic integration into bacterial genomes [154,155,156]. Prophages are widely distributed across various bacterial taxa, including *Lactobacillus* spp. [156], *Salmonella* spp. [157], and *Streptococcus* spp. [158]. Although phages exhibit specificity for bacterial hosts, they may indirectly influence the intestinal fungal community by altering bacterial composition and functional outputs due to the complex cross-kingdom interactions between bacteria and fungi within the gut ecosystem. Phage-induced lysis of bacterial hosts can disrupt metabolic networks that are crucial for fungal ecology. For example, SCFAs produced by commensal bacteria such as *Butyricoccus* and *Subdolicapsulum* possess antifungal properties against pathogens like *Aspergillus* spp. [75]. Phage-mediated depletion of SCFA-producing bacteria may lead to a reduction in these inhibitory metabolites, thereby altering fungal niche occupancy and proliferation dynamics. Conversely, the predation of phages on pathogenic bacteria, such as invasive *Salmonella* strains, has the potential to attenuate the antagonistic effect of these bacteria toward beneficial fungi. By selectively lysing bacterial competitors, phages can alleviate resource competition or suppression mediated by toxins. Consequently, this process fosters the expansion of commensal or symbiotic fungal populations [155]. The dual-regulatory capacity of phages prominently highlights their potential role as ecological mediators in the maintenance of gut microbiota equilibrium.

#### 4.5.2. Interactions Between Fungi

Similar to fungal–bacterial interactions, fungal interactions are predominantly characterized by antagonism [51]. Symbiotic fungi present in an animal’s gut stimulate antibody responses that safeguard the host from systemic and potentially fatal fungal infections. Antifungal secretory IgA (sIgA) induced by intestinal fungi can regulate co-colonization by covering fungal morphotypes linked to virulence, such as *C. albicans* [97]. Recently, a new strain belonging to the genus *Kazachstania*, specifically *K. weizmannii*, and its counterpart in the mouse intestine have been discovered to prevent the colonization of *C. albicans*, outcompete it during competitive seeding, and even expel it from stably colonized animals, demonstrating an inhibitory effect on *C. Parapsilosis* [159]. Conversely, *C. albicans* stimulates antifungal Th17 immunity through cross-reactivity with TH17 cells and suppresses the growth of other fungi [125]. A study reveals that when *C. albicans* is co-inoculated with other species, including *C. glabrata*, *C. parapsilosis*, *Issatchenkia orientalis*, and *Rhodotorula Mucilaginosa*, in the intestinal tracts of germ-free mice, it exhibits vigorous growth post-colonization, ultimately emerging as the dominant fungal species [59].

#### 4.5.3. Interactions Between Mycoviruse and Fungi

Mycoviruses, a group of viruses infecting fungi, are capable of inducing alterations in the host transcriptome profile via protein–protein interactions and the activation of anti-viral RNA silencing mechanisms within the fungus [160,161]. The majority of mycoviruses establish latent infections, signifying that they persist within the host fungus without eliciting conspicuous phenotypic changes. Some mycoviruses have the capacity to reduce the virulence of their fungal hosts, thereby diminishing their pathogenic potential. Conversely, a few mycoviruses may enhance the virulence of fungi, augmenting their ability to cause disease in hosts [161].

In the context of animal gut fungi, although the limited research on the direct influence of mycoviruses on the abundance of intestinal fungi, their effects on fungal phenotypes and growth can offer valuable insights. FodV1, a virus identified in isolate 116 (116V^+^) of *Fusarium oxysporum* f. sp. dianthi, induces remarkable phenotypic changes, such as altered vegetative growth and reduced virulence of *F. oxysporum* species [162]. *S. cerevisiae* strains have been confirmed as the host for helper L-A type totiviruses and satellite M double-stranded RNA (dsRNAs) associated with the killer phenotype [163]. Recently, a large-scale screening aimed at determining the diversity of dsRNA viruses in *S. cerevisiae* has identified multiple novel viruses from the family *Partitiviridae* [164]. This family was previously found within the Basidiomycota and Ascomycota fungi [165].

#### 4.5.4. Interaction Between Parasite and Fungi

Numerous studies have convincingly demonstrated that parasitic infections exert a significant influence on the composition, diversity, and immune responses of the gut microbiota in animals. Simultaneously, microorganisms can directly affect the survival, colonization, and expulsion of parasites or, alternatively, exert indirect effects through the host’s immune responses [166,167,168,169,170,171]. Currently, the number of studies indicating that parasites inhabiting the animal gut directly impact the composition and abundance of the mycobiota remains limited. For example, in *Procolobus gordonorum* and *Papio cynocephalus*, *Strongyloides* is found to have a negative association, while *Trichuris* has a positive association with fungal richness [172]. However, as previously mentioned, the microbiota has a substantial impact on fungal composition. Thus, the indirect effects of parasites on the mycobiota can be inferred from their interactions with the microbiota, particularly with bacterial genera like *Lactobacillus* and *Bifidobacterium*, which exhibit the most robust correlations with fungal communities.

For instance, the abundance of *Lactobacillus* is positively correlated with infection by the murine intestinal nematode parasite *Heligmosomoides polygyrus*, as well as the enhancement of regulatory T cell (Treg) and Th17 responses [173]. *Hymenolepis diminuta* can reduce the abundance of *Lactobacillus* and induce dysbiosis during the early stage of probiotic colonization in rats following infection [174]. Infection with *Blastocystis* can lead to a decrease in beneficial bacteria such as *Bifidobacterium* and *Lactobacillus* [175]. Conversely, mouse samples infected with *Plasmodium yoelii 17XNL* exhibit higher relative abundances of *Lactobacillus* and *Bifidobacterium* [176]. Since *Lactobacillus* and *Bifidobacterium* are negatively correlated with most fungi, including yeasts such as *C. albicans* and *Rhodotorula mucilaginosa*, parasitic infections in animals can indirectly influence the composition of the mycobiota by altering the structure of the microbiota.

Evidently, there is currently a pressing need for more in-depth research on the direct impact of intestinal parasites on gut fungi in monogastric animals, aiming to achieve a more profound understanding of the relationship between intestinal parasites and fungi.

### 4.6. Environment

From the moment of birth, animals receive nourishment from breast milk, whereas fungi obtain nutrients from their surrounding environment. Both organisms are continually subjected to diverse environmental factors as they undergo development [177].

The rearing environment plays a significant role in shaping the intestinal fungal community of reared animals. Apart from breast milk, maternal skin, and feces, microorganisms from the surrounding environment can also play a pivotal role in determining the fungal communities in newborn piglets [6]. While no direct comparison is made between wild and captive giant pandas regarding their fungal compositions, studies on the field training of captive giant pandas indicate that pandas that have undergone a period of reintroduction exhibit higher abundance and diversity of intestinal fungi compared to those that have recently commenced wild training [54]. Moreover, a higher abundance of the phylum Ascomycota and the genus *Peniophorella* has been detected in domestic cats, while the phylum Basidiomycota and the genus *Pichia* are more prevalent in outdoor cats [58]. In donkeys, anaerobic fungi, such as those belonging to the phylum Neocallimastigomycota, are significantly more abundant compared to ponies and mules/hinnies [65]. This phenomenon can be ascribed to the evolutionary adaptation of donkeys to arid regions characterized by scarce grass and vegetation [178]. In such environments, the presence of fungi like Neocallimastigomycota, which possess strong cellulose-degrading capabilities [89], becomes essential for donkeys to efficiently utilize the limited plant resources. Additionally, seasonal variations are demonstrated to impact mycobiota [179]. For instance, the fungal abundance in elephant feces collected during the late rainy season is higher than that collected during the dry season [180]. Interestingly, seasonal variations in the microbial environment of swine barns are found [179], suggesting that such changes may affect the composition of mycobiota in captive animals by altering the barn’s microbial environment.

### 4.7. Host Sex and Genetics

Currently, existing research, though limited in scope, indicates that host sex and genetics play a role in influencing the composition of animal intestinal fungi. In outbred murine models, host genetics, dietary patterns, and sex significantly mold the fungal composition. Specifically, the dietary composition contributes to 33% of the phenotypic variance in the heritability of the gut mycobiota, with an additional 12% being attributable to sex differences [46].

Notably, the heritability estimates for fungal abundance and α-diversity of pigs remained relatively low, ranging from 0.15 to 0.28. This low range implies that genetic factors have a limited influence on these traits [11]. In hybrid Xiangcun pigs, the gut mycobiome and the abundance of genes associated with host-pathogen interactions display intermediate characteristics between the Taoyuan and Duroc parental lineages. This observation suggests the potential for vertical transmission of gut fungi through heterozygote inheritance [49].

Comparative analyses of Chenghua, Yorkshire, and Tibetan pigs have revealed substantial interspecies divergence in gut fungal communities. This divergence is particularly evident in the composition at the phylum-level (the ratio of Ascomycota to Basidiomycota) and genus-level taxa such as *Loreleia* and *Candida* [51]. Another study has demonstrated that the relative abundance of *Piromyces* in pony/donkey hybrids is significantly higher than that in either donkeys or ponies, while the abundance of anaerobic fungi is significantly greater in donkeys compared to the other two equine types [65]. These findings underscore the combined effects of host sex, genetics, and evolutionary lineage in shaping the composition of the mycobiota across mammalian species.

### 4.8. Fungal-Based Probiotics and Prebiotics

Beyond the previously described effects of probiotics like *Lacticaseibacillus* spp. and *Bifidobacterium* spp. on intestinal fungal abundance, prebiotics degraded by gut microbiota may exert both direct and indirect impacts on fungal communities. This is because they are capable of inducing alterations in gastrointestinal bacterial populations and metabolic profiles, modulating intestinal and systemic immune functions, and enhancing the diversity and stability of the gut microbiota, especially by promoting the growth of beneficial bacteria [181].

For example, oligofructose and inulin have been demonstrated to reduce the abundance of *C. albicans* in the intestines of mice challenged with this fungus [182]. *Lacticaseibacillus rhamnosus* SD4, *L. rhamnosus* SD11, and *L. rhamnosus* GG, along with their cell-free supernatants (CFS), have shown strong anticandidal activity [183]. Low concentrations of konjac glucomannan hydrolysate (GMH) also enhance the resistance of *Lactobacillus jensenii* against *C. albicans* [184]. In the presence of inulin-type fructans, prebiotics produced by *Lactobacillus paracasei* or *Lactobacillus plantarum* exhibit anti-candidal effects, rendering them potential candidates for the production of antifungal agents or antimicrobial compounds [185].

Various prebiotics derived from fungi play a crucial role in maintaining gut health and improving the structure of mycobiota. Although *C. albicans* can exacerbate enteritis in immunocompromised hosts by taking advantage of host immune deficiencies, it does not trigger spontaneous intestinal inflammation in immunocompetent hosts with intact gut immunity [61]. Interestingly, certain *Candida* species possess anti-inflammatory potential. For instance, two acyclic sesquiterpenoid metabolites (F4 and F5) produced by *C. metapsilosis* M2006B have been found to significantly alleviate murine colitis through the selective activation of the farnesoid X receptor (FXR), indicating their therapeutic and prophylactic potential for IBD [163].

*C. albicans* further modulates intestinal immunity by inducing IL-22 production in CD4^+^ T helper cells. This induction enhances goblet cell proliferation [165], strengthens the epithelial barrier function, and activates transcriptional programs associated with JAK/STAT signaling and DNA repair. These mechanisms jointly protect mice from intestinal injury, bacterial translocation, and DSS-induced colitis [5]. Genetically modified *S*. *cerevisiae* strains that produce lactate have been shown to attenuate colitis by enhancing monocarboxylic acid transporter-mediated lactate uptake in macrophages. This process suppresses the hyperactivation of the NLRP3 inflammasome and the downstream caspase-1 pathway, ultimately reducing the expression of proinflammatory cytokines [166]. Notably, a deficiency in *S. cerevisiae* is associated with decreased IL-10 levels, and its supplementation has been proposed as an anti-inflammatory strategy [167]. Comparative studies reveal that *S. cerevisiae* stimulation induces significantly higher anti-inflammatory IL-10 production than *C. albicans*, emphasizing its distinct immunomodulatory profile [104].

## 5. Effects of Fungi on Metabolism of Nutrients in Monogastric Animals

### 5.1. Carbohydrate Metabolism

Fungal involvement in carbohydrate metabolism encompasses both the direct participation of secreted enzymes in the degradation of carbohydrates and the indirect modulation of host metabolic pathways through various mechanisms.

The utilization of dietary fiber (DF) and the production of oligosaccharides in monogastric mammals are heavily influenced by carbohydrate-active enzymes (CAZymes) produced by gut microorganisms. Fungi secrete CAZymes that are not only more abundant but also exhibit higher activity compared to those secreted by bacteria, thereby playing a crucial role in the degradation of DF [61]. Members of four fungal phyla, Chytridiomycota, Blastocladiomycota, Neocallimastigomycota, and Cryptomycota, are capable of secreting CAZymes. Various CAZymes, including auxiliary activity (AA) enzymes, carbohydrate esterase (CE), glycoside hydrolases (GH), glycosyltransferases (GT), and polysaccharide lyases (PL), can effectively degrade the primary components of DF [186].

Various low-abundance fungal genera associated with dietary carbohydrate metabolism, such as *Saccharomycopsis*, *Mrakia*, *Wallemia*, and *Cantharellus*, are also linked to glucose and fructose concentrations, as well as β-D-glucosidase activity in colonic digesta [61]. Neocallimastigomycota, a dominant fungus in the hindgut of Mongolian horses, also possesses a strong ability to decompose cellulose [15]. Notably, *Candida dubliniensis* and *K. slooffae* are observed to enhance host metabolism. Specifically, *K. slooffae* can activate SIRT5 activity via 5′-methylthioadenosine metabolites, promoting intestinal epithelial glycolysis through lysine desuccinimidation, which increases ATP production and subsequently boosts SCFAs production [11,53,59,111,187], which serves as an energy source for intestinal cells, supporting normal intestinal function, and induce immunomodulation to prevent inflammation and pathogenic infections [44,135]. Furthermore, co-colonization of *C. albicans*, *C. glabrata*, *C. parapsilosis*, *Issatchenkia orientalis*, and *Rhodotorula mucilaginosa* is demonstrated to promote the citric acid cycle and butyrate production in mice [59]. In *C. albicans*, Tye7p and Gal4p serve as key regulatory factors that initiate glycolysis [81]. Considering the fact that *C. albicans* has the capacity to colonize the stomach, it holds the potential to promote carbohydrate metabolism within the gastric environment.

Although there is little direct evidence suggesting that intestinal fungi definitely produce SCFAs, limited research demonstrates a connection between intestinal fungi in pigs and SCFAs. For example, *Metschnikowia* exhibits a positive correlation with acetate, propionate, and butyrate, while *Tomentella* shows a positive correlation with both acetate and butyrate. Furthermore, *Loreleia* has a positive correlation with propionate concentration. Conversely, *Nephroma* and *Taiwanofungus* have negative correlations with acetate and propionate concentrations, respectively [51]. In addition, *K. slooffae* is found to be positively correlated with butyrate, acetate, and propionate [85].

Mycobiota can influence metabolism by producing specific molecules and regulating the function of bacterial communities. When *Candida* digests dietary starch, it releases monosaccharides that are subsequently fermented by *Prevotella* and *Ruminococcus*. These fermentation by-products are then further broken down into carbon dioxide and methane by methanogens like *Methanobrevibacter* species [48].

### 5.2. Lipid Metabolism

Despite the long-standing interest in oleaginous fungi, which are utilized in diesel fuel production and are associated with lipid metabolism and, by extension, animal obesity [188], the mechanisms by which mycobiota contribute to diet-induced obesity remain unclear. Nevertheless, the role of fungi in lipid metabolism is not thoroughly investigated.

Recent studies hint at a close relationship between fungi and the host’s lipid metabolism. Species belonging to *Thermomyces*, *Saccharomyces*, and *Cladosporium* from Ascomycota, along with *Microascaceae* sp., contribute to the development of obesity, whereas *Helotiales* sp. and *Drechslera* sp. seem to inhibit it. Additionally, Clec7a is implicated in promoting lipid deposition facilitated by fungi [23,45]. Recently, *C. parapsilosis* has been found to elevate free fatty acid content through lipase production, a significant factor in diet-induced obesity [136]. Conversely, *Malassezia* has the potential to combat obesity by secreting lipase and other enzymes essential for fatty acid metabolism, enabling the utilization of extracellular fatty acids [189]. *Aspergillus* spp. are acknowledged as significant sources of acidic lipases, which have been designated as GRAS (generally recognized as safe) by the US Food and Drug Administration (FDA) and are intimately linked to lipid metabolism [82]. Moreover, the lipase secreted by *Phialemonium curvatum* maintains high activity even when the *pH* is 3 [83]. These fungi are likely to contribute to promoting the digestion and metabolism of fats in the stomach.

Bile acids (BAs) are crucial in lipid metabolism. Upon secretion into the intestinal lumen, they undergo conversion into secondary BAs by mycobiota [190]. Specific filamentous fungi, such as *Penicillium* spp. and *Aspergillus* spp., possess the ability to produce structurally unique bile acid derivatives, including hydroxylated and oxidized bile acid molecules, through various chemical modifications like hydroxylation, dehydrogenation, and cyclization [191]. Additionally, *Fusarium* spp. and *C. albicans* enhance the levels of cholic acid, deoxycholic acid, and hyocholic acid, collectively facilitating lipid metabolism [23,116].

### 5.3. Protein Metabolism

Research on fungi, including yeast and *Aspergillus oryzae*, boasts a lengthy history, particularly in in vitro experiments involving protein feed fermentation [192]. Studies reveal that the extracellular protease secreted by *Aspergillus* species present in the animal intestine exhibits exceptional protein catabolic capabilities [193]. While research on the role of fungi in protein metabolism within the animal gut is limited, existing studies suggest that certain fungi are indeed involved in this process. For example, *K. slooffiae* in the porcine intestine may interact with intestinal bacteria to influence host carbohydrate and protein metabolism [187]. The in vitro assays demonstrate *K. slooffiae*’s ability to utilize urea, ammonium sulfate, peptides, and amino acids to produce ethanol, formic acid, and oligopeptides, and it produces more lysine than *S. cerevisiae* [85]. Furthermore, *Neoascochyta europaea* is found to enhance the degradation of leucine and methionine while facilitating tryptophan synthesis [48]. *S. cerevisiae* can inhibit aspartate aminotransferase, alanine aminotransferase, gamma-glutamyl transferase, and other enzymes linked to liver disease development, thereby preventing metabolic liver diseases [111].

### 5.4. Vitamins and Minerals

Intestinal fungi play a critical role in vitamin and mineral metabolism, with their diversity and function exerting a profound influence on the absorption and processing of micronutrients within the host. A number of gut fungi possess the ability to synthesize various vitamins. For instance, *S*. *cerevisiae* has been experimentally demonstrated to participate in the de novo synthesis of thiamine (B1) [194,195], biotin (B7) [196], pantothenic acid (B5) [197,198], and nicotinic acid (B3) [199,200] and shows potential for the synthesis of vitamin A [201], E [202], and K [203]. In filamentous fungi such as *Aspergillus nidulans*, pimeloyl-CoA, a precursor in biotin synthesis, is generated in peroxisomes via the β-oxidation cycle [196]. These vitamins are indispensable for cellular metabolism, growth, and neurological functions in animals.

In the context of mineral metabolism, numerous gut fungi secrete chelators, such as siderophores, to bind metal ions [204,205], thereby altering the bioavailability of minerals. Certain fungi are also capable of modulating the expression of metal transporters in IECs, thus regulating the host’s absorption of these minerals [206,207,208]. Fungal fermentation, especially by yeasts, can influence the utilization of minerals in the gut, either promoting or inhibiting the absorption of calcium, iron, and others. For example, the co-fermentation of soymilk by *Saccharomyces boulardii* and *Lactobacillus* spp. enhances the bioavailability of calcium and magnesium while reducing the bioavailability of iron [209]. In *S*. *cerevisiae*, the *ECM27* gene, which encodes a Na^+^/Ca^2+^ exchanger, regulates the influx of calcium from extracellular spaces and its release from intracellular stores during membrane stress [210]. Iron metabolism in *S. cerevisiae* is regulated by transcription factors Aft1/Aft2 and Yap5, which respond to low and high iron levels, respectively. Under iron-deficient conditions, Aft1/Aft2 activate genes involved in iron uptake and transport to maintain iron homeostasis [211,212]. In *Aspergillus fumigatus*, the *AcuM* gene modulates iron acquisition under iron-scarce conditions by repressing *SreA* and stimulating *HapX* [213]. *A. fumigatus* produces extracellular siderophores for iron uptake and intracellular ferritin-like proteins for iron storage, alleviating iron toxicity in the host [214]. Additionally, the catalytic subunit PpzA of protein phosphatase Z (PPZ) in *A. fumigatus* has been associated with iron assimilation [215].

Yeast cells can assimilate selenium in both organic and inorganic forms. During intracellular selenium metabolism, processes, including oxidation, reduction, methylation, and selenoprotein synthesis, occur, and selenium-enriched yeast represents a highly bioavailable form of this element [216]. In *C. albicans*, the zinc transporter Zrt2 (a member of the ZIP family) is critical for zinc uptake and growth under acidic *pH* conditions. Deletion of PRA1, a zinc-binding protein in *C. albicans*, impairs fungal zinc sequestration and the host’s zinc utilization, specifically preventing host cell damage in zinc-deficient environments [217]. Pra1, a 299-amino acid zincophore secreted by *C. albicans*, binds Zn(II) and transports it to the transmembrane zinc transporter Zrt1, playing a key role in fungal pathogenicity during infections [218].

Minerals usually play a pivotal role in the metabolism of the host. For instance, iron, a redox-active element, serves as a critical cofactor in metabolic pathways such as respiration, DNA synthesis, and translation. Iron deficiency disrupts glucose metabolism, amino acid biosynthesis, and lipid biosynthesis [219]. Selenium, a trace element essential for biological functions, is a component of selenoproteins and antioxidant enzymes such as glutathione peroxidase (GPx), thioredoxin reductase (TRxR), and iodothyronine deiodinase (DIO), which protect cells from oxidative damage [220]. This intricate interplay between gut fungi and micronutrient metabolism highlights their potential as modulators of host nutrition and health, justifying further in-depth exploration of their therapeutic and functional roles.

## 6. Conclusions

**Figure 2:**
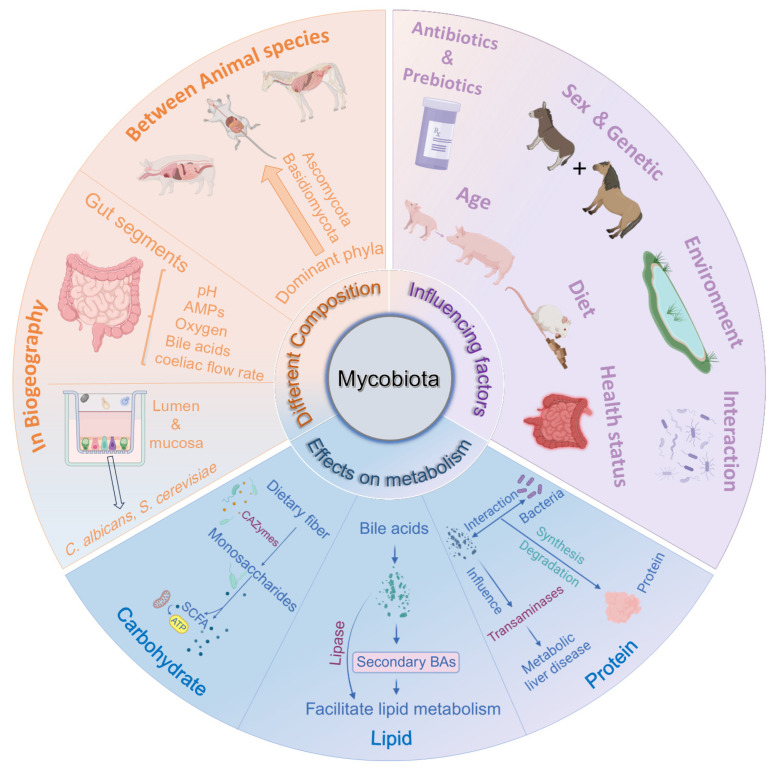
The composition and distribution of gut mycobiota: variations across animal species and intestinal segments, influenced by multiple factors, and with significant metabolic impacts on host carbohydrate, lipid, and protein metabolism.

To date, the composition and distribution of mycobiota in the animal intestine have been initially characterized through the application of microbiomics, transcriptomics, metabolomics, and macrogenomics. However, there remains a notable gap in research focused on the species-level composition and distribution of mycobiota. Most studies examining the factors influencing mycobiota composition changes and their role in animal intestinal metabolism primarily established correlations, with only a limited number demonstrating causality. This underscores the incomplete understanding of this significant microbiota group in the animal gut (Figure 2). Therefore, more in-depth mechanistic studies are imperative to bridge the gap between current correlation findings and causative relationships. Despite a systematic mapping of these relatively under-explored aspects, our analysis charts a course for the transition from merely observational correlations to practical therapeutic applications. Targeted mechanistic investigations into fungal metabolism, immune modulation pathways, and quantitative aspects will not only elucidate their causal functions in gut metabolism but also empower the precise manipulation of the mycobiota for personalized disease interventions. The framework established herein promotes intestinal fungi from being overlooked commensals to becoming potential therapeutic targets, underscoring their potential for personalized metabolic regulation and enhancing disease prevention and treatment strategies.

## Figures and Tables

**Table 1:** Predominant intestinal fungi and their relative abundance across various animal species at different classification levels.

Animals		Relative Abundance	
	Phyla	Genus	Species
Mouse	- Ascomycota (~59.0–97.0% [45,46,47]) - Basidiomycota (~2.0–12.0% [45,46]) - Chytridiomycota [13] - Zygomycota [13]	- *Penicillium* (~53.3% [46]) - *Aspergillus* (~8.4% [46]) - *Candida* (~7.7% [46,48]) - *Saccharomyces* [48]	- *Candida albicans* (~22.0–38.0% [13,48]) - *Saccharomyces cerevisiae* (~40.0% [48])
Pig	- Ascomycota (~24.3–68.0% [49,50,51]) - Basidiomycota (~19.1–73.9% [50,51]) - Mucoromycota (~0.4% [49,52]) - Microsporidia (~0.1% [49,52]) - Chytridiomycota (~0.03% [49,52])	- *Kazachstania* (~23.8% [49,52]) - *Mucor* (~6.9% [49,52]) - *Anaeromyces* [49] - *Piromyces* [49] - *Neocallimastix* [49] - *Naganishia* (~80.2% [52]) - *Rhodotorula* (~9.7% [52]) - *Mortierella* (~8.4% [52]) - *Candida* (~1.8% [52])	- *Kazachstania slooffiae* (~72.3–76.6% [11,53]) - *Kazachstania telluris* (>78.0% [47]) - *Kazachstania bovina* (~6.4% [11])
Giant Panda	- Basidiomycota (~7.6–33.2% [37,54]) - Ascomycota (~75.5% [37]) - Glomeromycota (~5.4% [37])	- *Fusarium* (~22.6% [37]) - *Brettanomyces* (~9.6% [37]) - *Oidiodendron* (~9.1% [37]) - *Trimmatostroma* (~6.2–19.6% [55])	- *Fusarium oxysporum* (~17.5% [37]) - *Fusarium proliferatum* (~11.2% [37]) - *Brettanomyces custersianus* (~11.0% [37])
Equines	- Ascomycota (~55.0–73.1% [20,41]) - Basidiomycota (~0.9–12.5% [20,41]) - Mucoromycota (~7.9% [41]) - Neocallimastigomycota (~2.3–21.3% [20,41])	- *Cladosporium* (~2.4% [15]) - *Cryptococcus* (~2.3% [15])	
Rabbit	- Ascomycota (~74.7% [41]) - Basidiomycota (~0.3% [41])		- *Cyniclomyces guttulatus* (~95.2% [21])
Elephant	- Ascomycota (~80.7–95.1% [41]) - Basidiomycota (~4.4–14.0% [41])	- *Preussia* (~2.3–19.2% [40]) - *Aureobasidium* (~0.7–25.4% [40]) - *Didymella* (~5.0–15.3% [40]) - *Alternaria* (~2.2–12.0% [40])	- *Trichoderma aureoviride* [56] - *Fusarium equiseti* [56]
Dog	- Ascomycota (~63.2–100.0% [38,57]) - Basidiomycota (~0.0–36.8% [38,57]) - Glomeromycota [38] - Zygomycota [38]	- *Nakaseomyces* (~76.7% [38])	- *Candida castellii* [38] - *Candida natalensis* [57] - *Catenulostroma abietis* [57]
Cat	- Ascomycota [38] - Basidiomycota [58]	- *Saccharomyces* (~58.3% [38]) - *Aspergillus* (~11.0% [38]) - *Peniophorella* [58] - *Pichia* [58]	

## Data Availability

No new data were created in this paper, which serves as a comprehensive review of existing knowledge.
